# Rescue Transcatheter Aortic Valve Implantation for Severe Native Aortic Regurgitation in a Young Patient with Hypertrophic Obstructive Cardiomyopathy and Cardiogenic Shock: Review of the Literature and Case Presentation

**DOI:** 10.3390/life16091503

**Published:** 2026-09-09

**Authors:** Catalina Andreea Parasca, Dan Deleanu, Pavel Platon, Crina Ioana Radulescu, Mihai Stefan, Ruxandra Oana Jurcut, Daniela Carmen Filipescu, Vlad Anton Iliescu

**Affiliations:** 1 Faculty of Medicine, Carol Davila University of Medicine and Pharmacy, 050474 Bucharest, Romaniavlad.iliescu@umfcd.ro (V.A.I.); 2 Department of Cardiovascular Surgery, “Prof. Dr. C.C. Iliescu” Emergency Institute for Cardiovascular Diseases, 022328 Bucharest, Romania; 3 Department of Interventional Cardiology, “Prof. Dr. C.C. Iliescu” Emergency Institute for Cardiovascular Diseases, 022328 Bucharest, Romania; 4 Department of Anesthesiology and Intensive Care, “Prof. Dr. C.C. Iliescu” Emergency Institute for Cardiovascular Diseases, 022328 Bucharest, Romania; 5 Department of Cardiology, “Prof. Dr. C.C. Iliescu” Emergency Institute for Cardiovascular Diseases, 022328 Bucharest, Romania

**Keywords:** aortic regurgitation, septal myectomy, transcatheter aortic valve implantation, cardiogenic shock, hypertrophic cardiomyopathy

## Abstract

Transcatheter aortic valve implantation (TAVI) for pure native aortic regurgitation (AR) remains technically challenging, primarily because absent annular calcification limits prosthesis anchoring. We reviewed contemporary evidence on TAVI for native AR, focusing on devices, procedural performance, complications, and outcomes. A comprehensive PubMed search identified 371 articles, of which 28 studies comprising 3282 patients met the inclusion criteria; notably, 64.3% were published from 2023 onward, reflecting the rapidly expanding evidence in this field. Conventional off-label devices were evaluated in 60.7% of studies and dedicated AR devices in 35.7%, while 3.6% directly compared both strategies. Technical/device success ranged from 72% to 100%, generally exceeding 85–90% in contemporary series, while 30-day mortality ranged from 0% to 23%. To illustrate the technical challenges and expand this evidence, we present a 24-year-old man with MYH7-associated hypertrophic obstructive cardiomyopathy and severe post-myectomy AR complicated by biventricular failure, cardiogenic shock, and multiorgan dysfunction. Given prohibitive surgical risk, rescue transfemoral TAVI was performed. Despite embolization of the first balloon-expandable prosthesis, a second valve was successfully implanted using an individualized anchoring strategy. At 1 year, the patient was in NYHA class I with biventricular recovery. TAVI represents an evolving alternative for carefully selected patients with severe native AR and prohibitive surgical risk.

## 1. Introduction

Hypertrophic cardiomyopathy (HCM) is a genetically determined myocardial disorder characterized by unexplained left ventricular hypertrophy, myocyte disarray, myocardial fibrosis, and an increased risk of arrhythmias, heart failure, and sudden cardiac death [1,2]. The estimated prevalence is approximately 1 in 500 individuals, making it one of the most common inherited cardiovascular diseases [1,2]. Pathogenic variants involving sarcomeric genes account for most familial cases, with MYH7 and MYBPC3 representing the most frequently implicated genes [3,4]. Mutations in MYH7 have been associated with earlier disease onset, severe phenotypic expression, and adverse clinical outcomes in some families [5].

In patients with hypertrophic obstructive cardiomyopathy (HOCM), surgical septal myectomy remains the gold standard treatment for severe symptomatic left ventricular outflow tract obstruction refractory to medical therapy [6]. Long-term outcomes are generally excellent; however, complications involving the mitral valve, conduction system, and, less frequently, the aortic valve may occur [7]. Procedure-related AR is observed in approximately 2% of patients undergoing transaortic septal myectomy, with approximately half requiring unplanned aortic valve procedures at the time of surgery [8].

Severe native aortic regurgitation in a young patient with previous myectomy presents a challenging therapeutic scenario. Surgical valve replacement may carry excessive risk in patients with advanced ventricular dysfunction and persistent hypoperfusion causing multiorgan failure. In recent years, TAVI has emerged as an alternative treatment for selected patients with pure native aortic regurgitation, although technical challenges remain because of the absence of annular calcification and the increased risk of prosthesis migration [9,10].

We report a complex case of MYH7-associated HOCM complicated by severe aortic regurgitation, progressive HF and ultimately cardiogenic shock resolved by successful rescue TAVI. We also provide a comprehensive review of the literature of the evidence regarding transcatheter treatment of pure native aortic regurgitation, post-myectomy aortic regurgitation, TAVI in cardiogenic shock, predictors of ventricular recovery following valve intervention, and lifetime management strategies in young patients.

## 2. Case Presentation

A 24-year-old man with a complex history of inherited cardiovascular disease, followed in our center’s genetic cardiomyopathy program, was admitted in March 2025 with severe decompensated heart failure complicated by hepatic and renal dysfunction due to systemic congestion. His presentation occurred in the context of a highly malignant familial cardiomyopathy phenotype and previous surgical treatment for obstructive HCM.

### 2.1. Family History and Initial Diagnosis

The patient’s family history was notable for a highly malignant hereditary cardiomyopathy phenotype. His brother had been diagnosed with HCM and died suddenly at 22 years of age. His father also suffered sudden cardiac death despite having undergone prophylactic ICD implantation. An uncle died suddenly at a young age, while an 11-year-old niece had already been diagnosed with HCM. The clustering of affected family members across several generations suggested a highly penetrant inherited disease with substantial arrhythmic risk. The patient was diagnosed with HOCM in 2015 after evaluation for recurrent episodes of non-sustained ventricular tachycardia and atrial arrhythmias. Genetic testing identified a pathogenic variant in the MYH7 gene (p.Arg719Trp), confirming the diagnosis of sarcomeric HCM. At the time of diagnosis, echocardiography demonstrated asymmetric septal hypertrophy associated with significant left ventricular outflow tract obstruction. Given the severity of obstruction and symptoms, the patient was referred to surgical treatment.

### 2.2. Surgical History

At the age of 14 years, the patient underwent extended transaortic septal myectomy combined with mitral valve repair and secondary chordal resection. While the operation resulted in complete relief of dynamic left ventricle outflow tract (LVOT) obstruction, it was associated with partial aortic cusp injury resulting in moderate AR. Additionally, the postoperative course was complicated by complete atrioventricular block, necessitating implantation of a dual-chamber ICD for permanent pacing and primary prevention of sudden cardiac death. Initially, the patient experienced substantial symptomatic improvement and remained clinically stable for several years.

### 2.3. Clinical Deterioration

**Figure 1:**
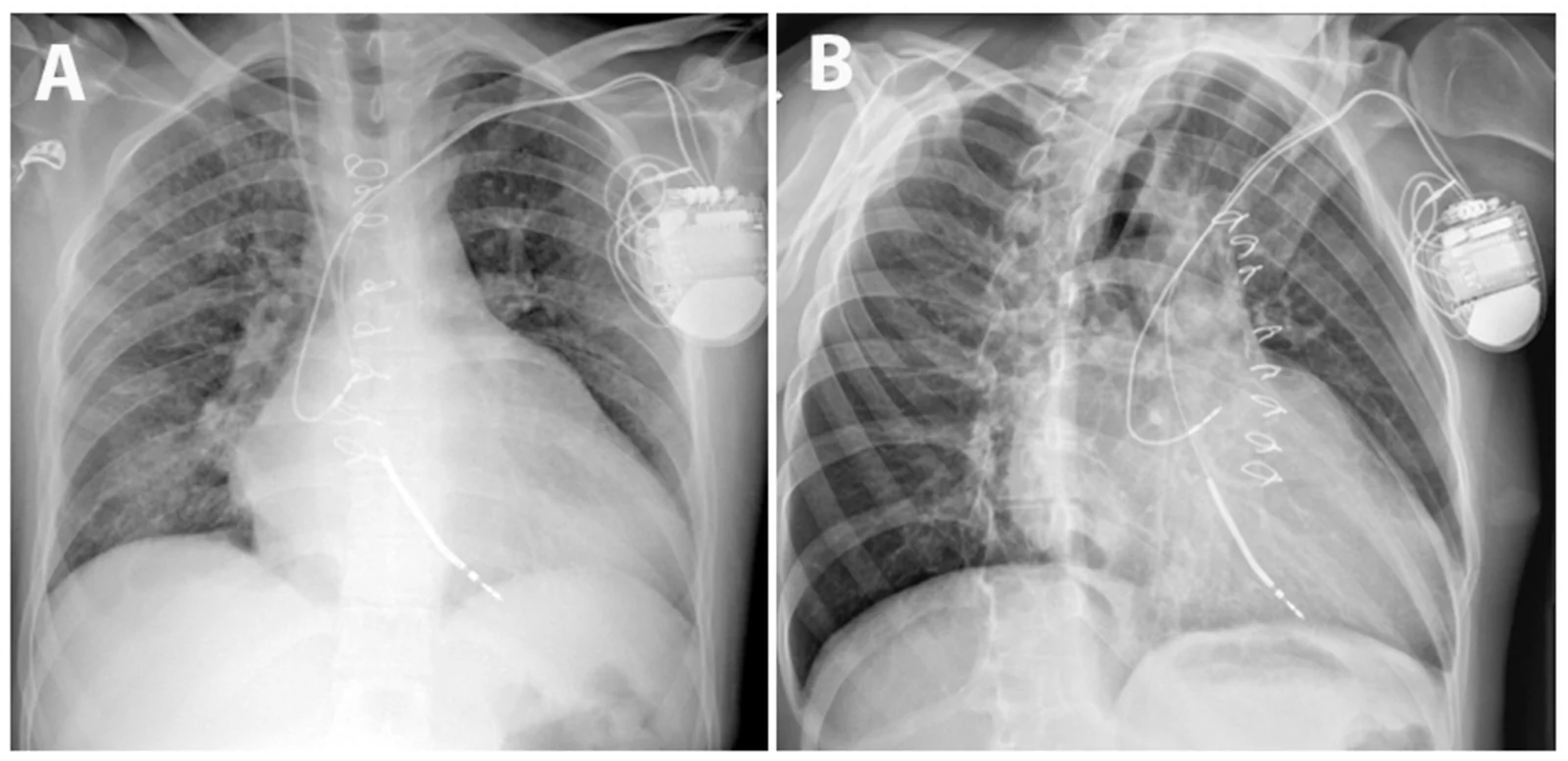
Preprocedural chest radiographs. (**A**) Frontal chest radiograph demonstrating cardiomegaly with pulmonary vascular congestion in the setting of acute decompensated heart failure. (**B**) Right anterior oblique (RAO) 60° projection obtained showing the left-sided dual-chamber implantable cardioverter-defibrillator (ICD) and median sternotomy wires from previous cardiac (septal myectomy).

Seven years after surgery the patient’s clinical condition gradually deteriorated, resulting in multiple admissions for decompensated heart failure over a period of six months. In March 2025 he was readmitted with decompensated heart failure accompanied by severe hepatic dysfunction, cholestatic jaundice, renal impairment, and systemic congestion. Chest X-ray in the emergency department also showed signs of pulmonary congestion (Figure 1). Laboratory tests revealed: NT-proBNP approximately 7500 pg/mL; total bilirubin 21 mg/dL with direct bilirubin 17.5 mg/dL; elevated transaminases, acute kidney injury and severe hypoalbuminemia. Comprehensive infectious and autoimmune investigations were negative.

Transthoracic echocardiography (TTE) identified severe AR caused by marked restriction of the right coronary cusp, possibly due to progressive fibrosis of the previously injured cusp, resulting in failure of cusp coaptation. Additional findings included severe left atrial (LA) enlargement, spontaneous echo contrast within the LA, suspected LA appendage thrombus, mild left ventricular systolic dysfunction, mild-to-moderate mitral regurgitation and mild tricuspid regurgitation.

### 2.4. Pre-Procedural Status

**Figure 2:**
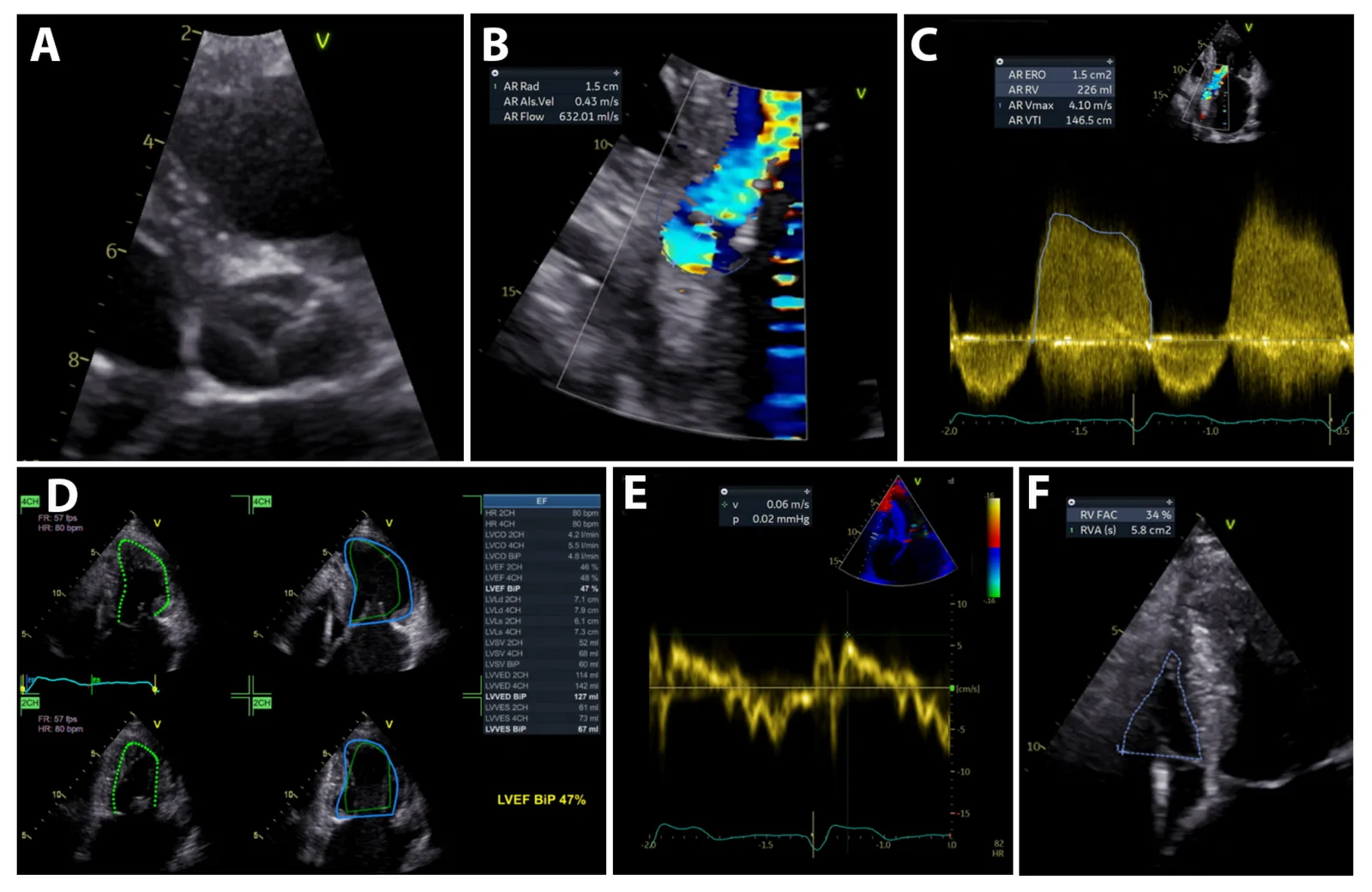
Transthoracic echocardiography showing modified tricuspid aortic valve with right cusp restriction (**A**) and severe regurgitation (**B**,**C**); Significant left ventricle dilatation with reduced ejection fraction (**D**) and severe right ventricular dysfunction (**E**,**F**).

Despite guideline-directed medical therapy, the patient developed progressive heart failure and worsening functional capacity over the following years, due to progression of AR from moderate to severe, chamber enlargement and deterioration of LV systolic function, as shown by serial echocardiographic examinations during follow-up. By March 2025, the patient experienced significant worsening heart failure, laboratory findings demonstrated NT-proBNP 16,933 pg/mL, Lactate 3.9 mmol/L, total bilirubin 11.2 mg/dL, hyponatremia (129 mmol/L), elevated liver enzymes and anemia, while echocardiography showed severe AR, moderate mitral and tricuspid regurgitations, right ventricular (RV) dysfunction, pulmonary hypertension and, significant LV dilatation with reduced ejection fraction (EF) (Figure 2). Clinically, the patient experienced recurrent episodes of systemic hypoperfusion with subsequent worsening heart failure. At this point, conventional surgical aortic valve replacement was considered extremely high risk taking into account surgical redo in the acute setting of cardiogenic shock with severe RV dysfunction, reduced EF and multiorgan failure.

### 2.5. Heart Team Decision, Planning and TAVI Procedure

The case was discussed by a multidisciplinary Heart Team consisting of specialists in heart failure, imaging, cardiac surgery, congenital and structural heart disease, and interventional cardiology. Given the patient’s condition at the time, cardiogenic shock due to severe biventricular dysfunction with multiorgan failure, previous transaortic septal myectomy, and prohibitive risk associated with redo surgery, transfemoral TAVI was considered the only feasible therapeutic option at that moment. The potential benefits and procedural risks, including the off-label use of a conventional transcatheter heart valve for pure native aortic regurgitation in the absence of a dedicated device available in our center, as well as the uncertain long-term durability and implications for lifetime valve management, were carefully considered. The possibility of subsequent surgical or transcatheter intervention, as well as the potential role of TAVI as a bridge to recovery allowing another surgery in the future. In the case of persistent ventricular dysfunction, advanced heart failure therapies such as ventricular assist devices or heart transplantation were considered. As part of the shared decision-making process, all these scenarios have been largely discussed with the patient and his family.

**Figure 3:**
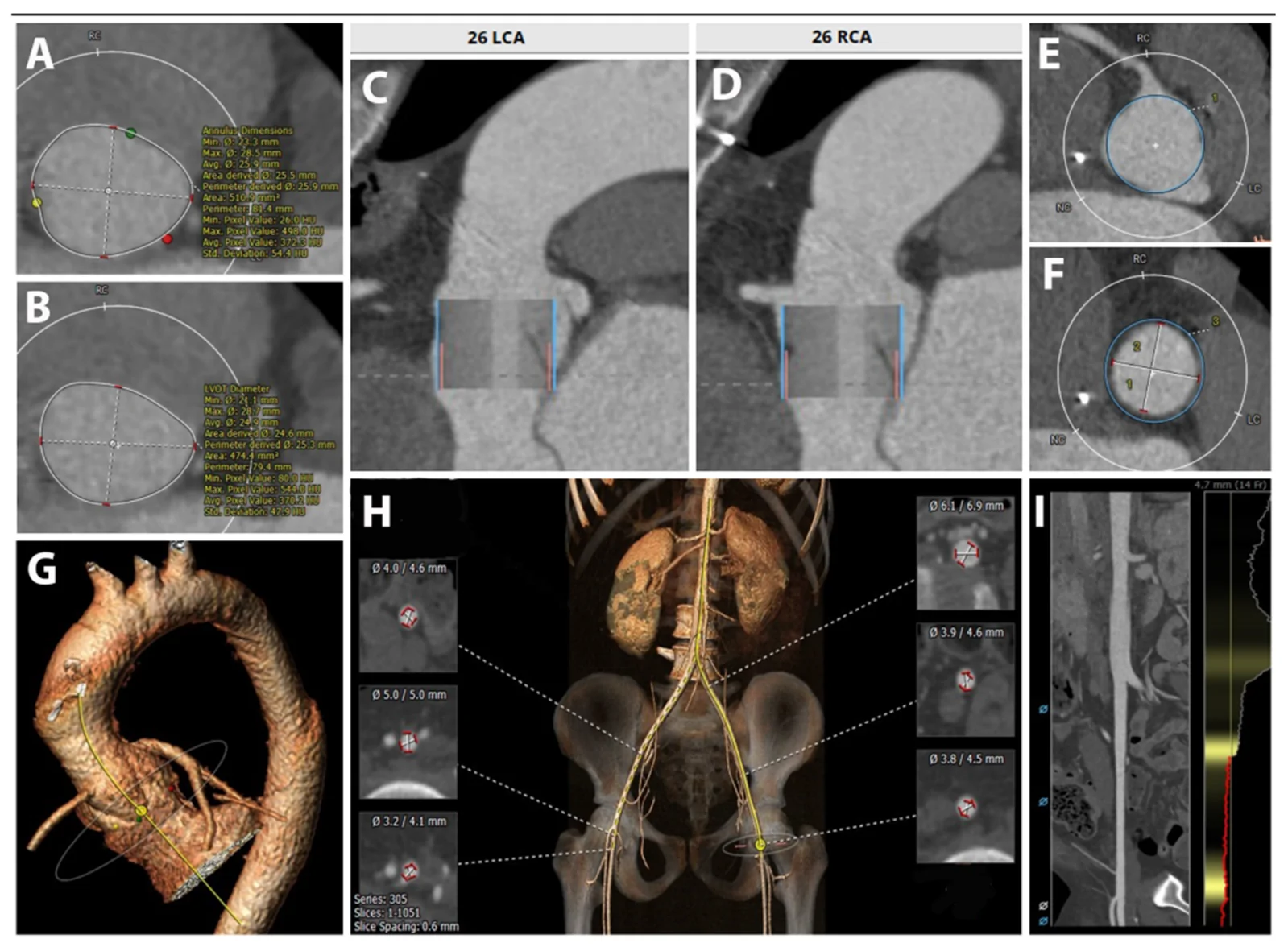
Multidetector computed tomography (MDCT) assessment for preprocedural TAVI planning. CT analysis included annular and LVOT sizing (**A**,**B**), coronary ostial height assessment (**C**,**D**), sinuses of Valsalva and sinotubular junction evaluation (**E**,**F**) in relation with valve projection; three-dimensional reconstruction of the aortic root for assessment of the optimal fluoroscopic implantation angle (**G**); assessment of iliofemoral access with vessel diameter measurements and curved multiplanar reconstruction confirming suitability for transfemoral TAVI (**H**,**I**).

Pre-procedural planning included ECG-gated computed tomography to assess annular dimensions, aortic root anatomy, and procedural feasibility. The pre-procedural CT demonstrated an annular area of 510 mm^2^ (area-derived diameter, 25.5 mm) and an LVOT area of 474 mm^2^ (area-derived diameter, 24.7 mm). CT also revealed a small sinotubular junction (23 mm) and ascending aorta (29 mm), with the latter previously surgically sutured during the prior operation, resulting in a relatively constrained and surgically altered aortic root. Based on the annular measurements, evaluation and virtual simulation were initially performed using a 26 mm balloon-expandable valve (BEV) (Edwards SAPIEN 3 Ultra RESILIA transcatheter heart valve; Edwards Lifesciences, Irvine, CA, USA) (Figure 3).

**Figure 4:**
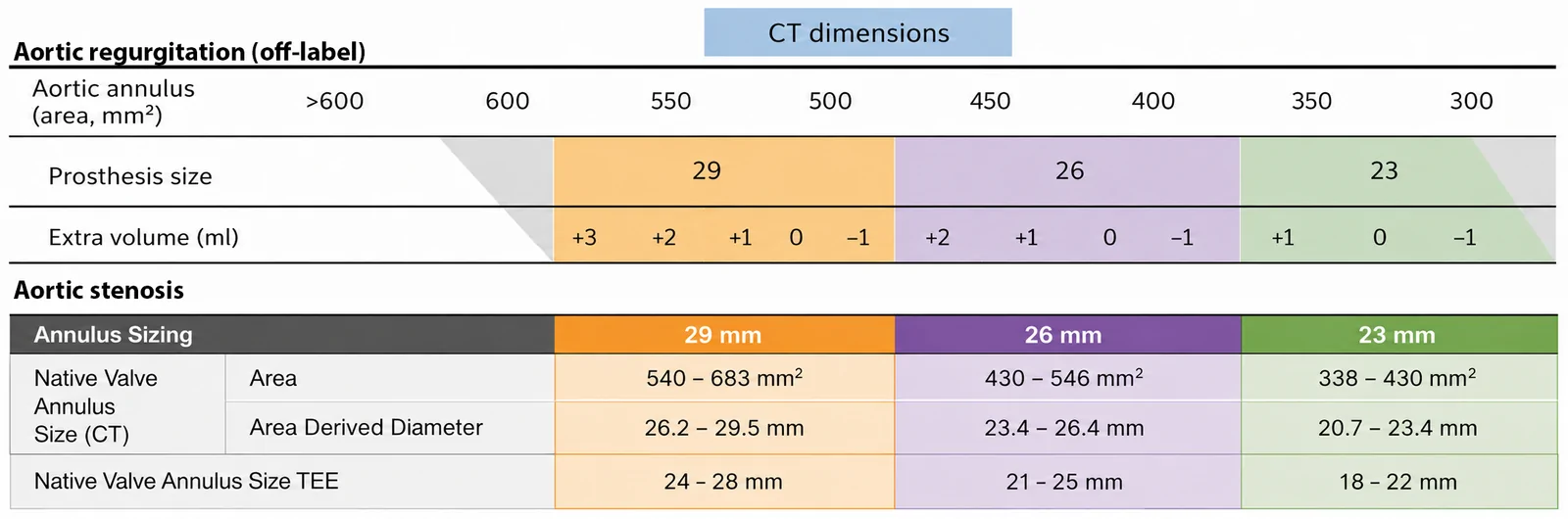
Valve selection and sizing strategy for balloon expandable transcatheter aortic valve implantation in pure native aortic regurgitation.

A BEV was preferred because its controlled deployment and high radial force were considered advantageous for anchoring in the setting of non-calcified native aortic regurgitation. Given the absence of annular calcification, intentional prosthetic oversizing was considered necessary to enhance anchoring and reduce the risk of valve embolization. Although the 26 mm prosthesis was anatomically compatible with the measured annular dimensions, it would provide only approximately 7% area oversizing, whereas the 29 mm valve would provide approximately 34% oversizing. Therefore, the 29 mm BEV (Edwards SAPIEN 3 Ultra RESILIA transcatheter heart valve; Edwards Lifesciences, Irvine, CA, USA) was ultimately selected to maximize prosthetic anchoring, with careful consideration of the potential interaction between the oversized prosthesis and the relatively small, previously surgically altered aortic root (Figure 4).

After informed consent was obtained from the patient and his family, the procedure was performed under general anesthesia and TEE guidance. A 29 mm Edwards SAPIEN 3 Ultra RESILIA transcatheter heart valve (Edwards Lifesciences, Irvine, CA, USA) was implanted through a right transfemoral approach. During the first implantation attempt, the valve was intentionally crimped in a higher position on the balloon to preferentially expand the ventricular portion of the frame while minimizing outflow flaring and protecting the relatively constrained and surgically altered aortic root. This resulted in insufficient frame expansion during deployment under rapid pacing, with inadequate anchoring and subsequent valve embolization. The embolized valve was successfully captured with a balloon catheter and repositioned in the descending thoracic aorta, where it remained stable without causing flow limitation, aortic wall injury or requiring surgical retrieval.

**Figure 5:**
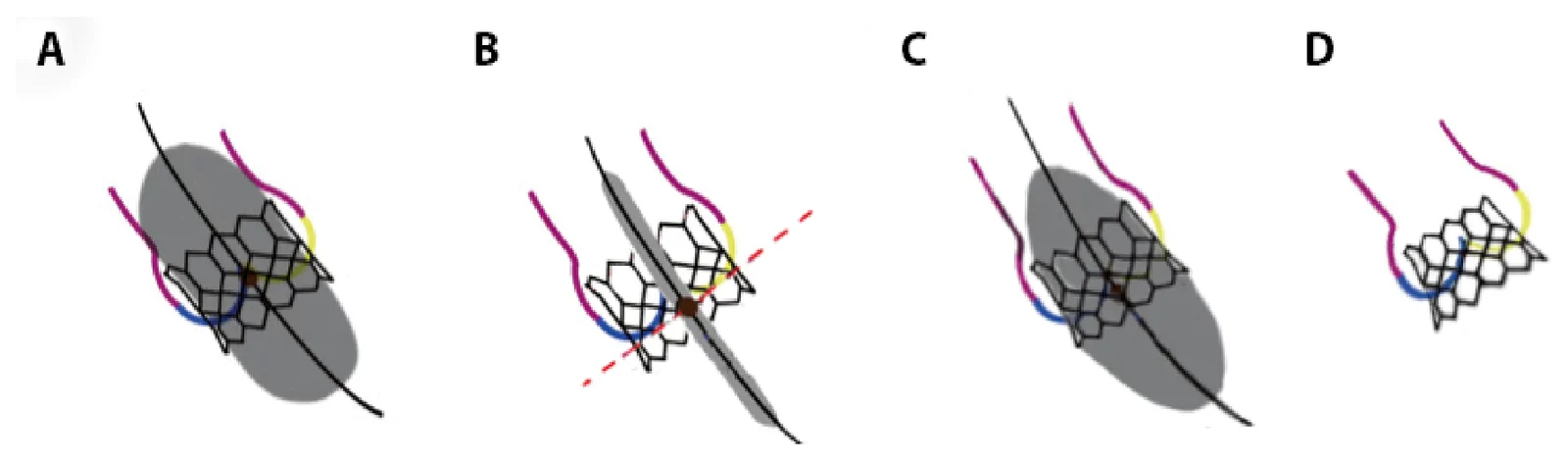
Modified implantation technique to improve prosthesis anchoring. (**A**) Standard valve position on the delivery balloon during valve deployment; (**B**,**C**) Ballon positioning and post dilatation strategy to limit outflow flaring and protect the previously operated aortic root while increasing stability by inflow flaring; (**D**) Valve position and flaring aspect.

A second 29 mm valve was then implanted using the standard balloon position but at a lower depth within the LVOT. Additional post-dilatation was performed to selectively flare the inflow portion of the stent frame, improving LVOT anchoring and resulting in a stable final prosthesis position (Figure 5). The improved stability was therefore attributed primarily to more adequate ventricular frame expansion and optimized inflow anchoring, while maintaining an acceptable relationship with the surgically altered aortic root.

**Figure 6:**
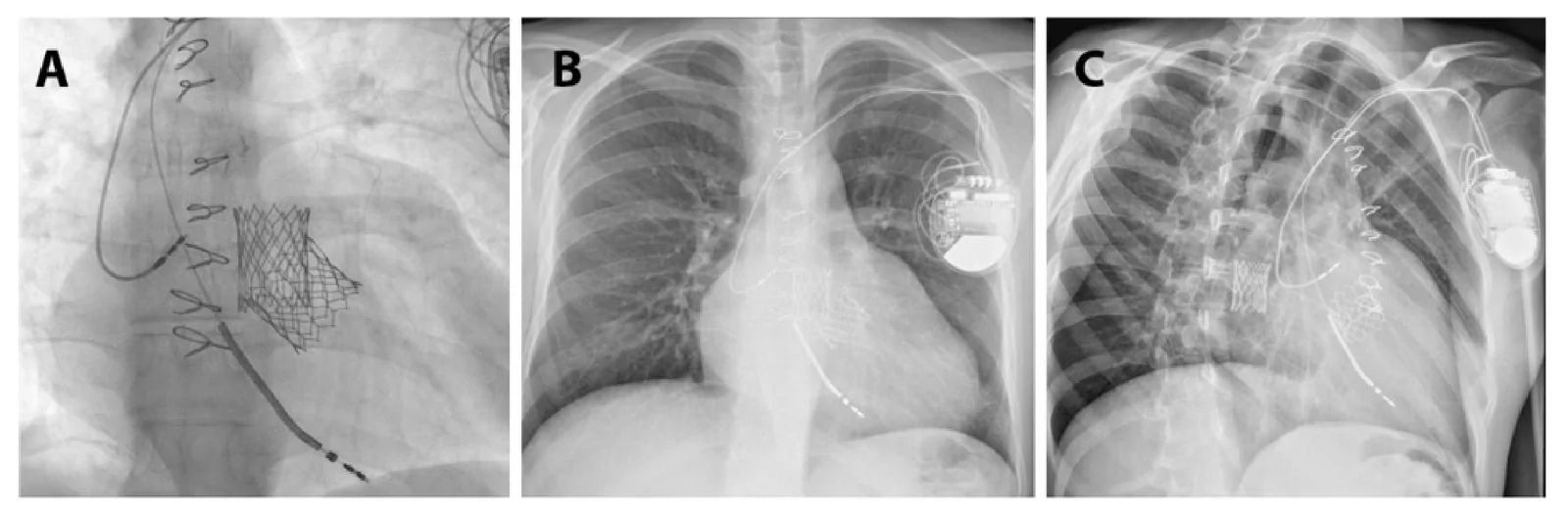
Imaging after transcatheter aortic valve implantation. (**A**) Final fluoroscopic image demonstrating successful implantation of the second Edwards SAPIEN 3 valve in the native aortic annulus after embolization of the first prosthesis to the descending thoracic aorta. (**B**) Frontal chest radiograph and (**C**) 60° right anterior oblique chest radiograph confirming stable valve position at follow-up.

Post-procedural imaging confirmed stable valve position with no significant paravalvular leak, acceptable transvalvular gradients and immediate hemodynamic improvement (Figure 6). No emergency surgical conversion was required.

### 2.6. Follow-Up

**Figure 7:**
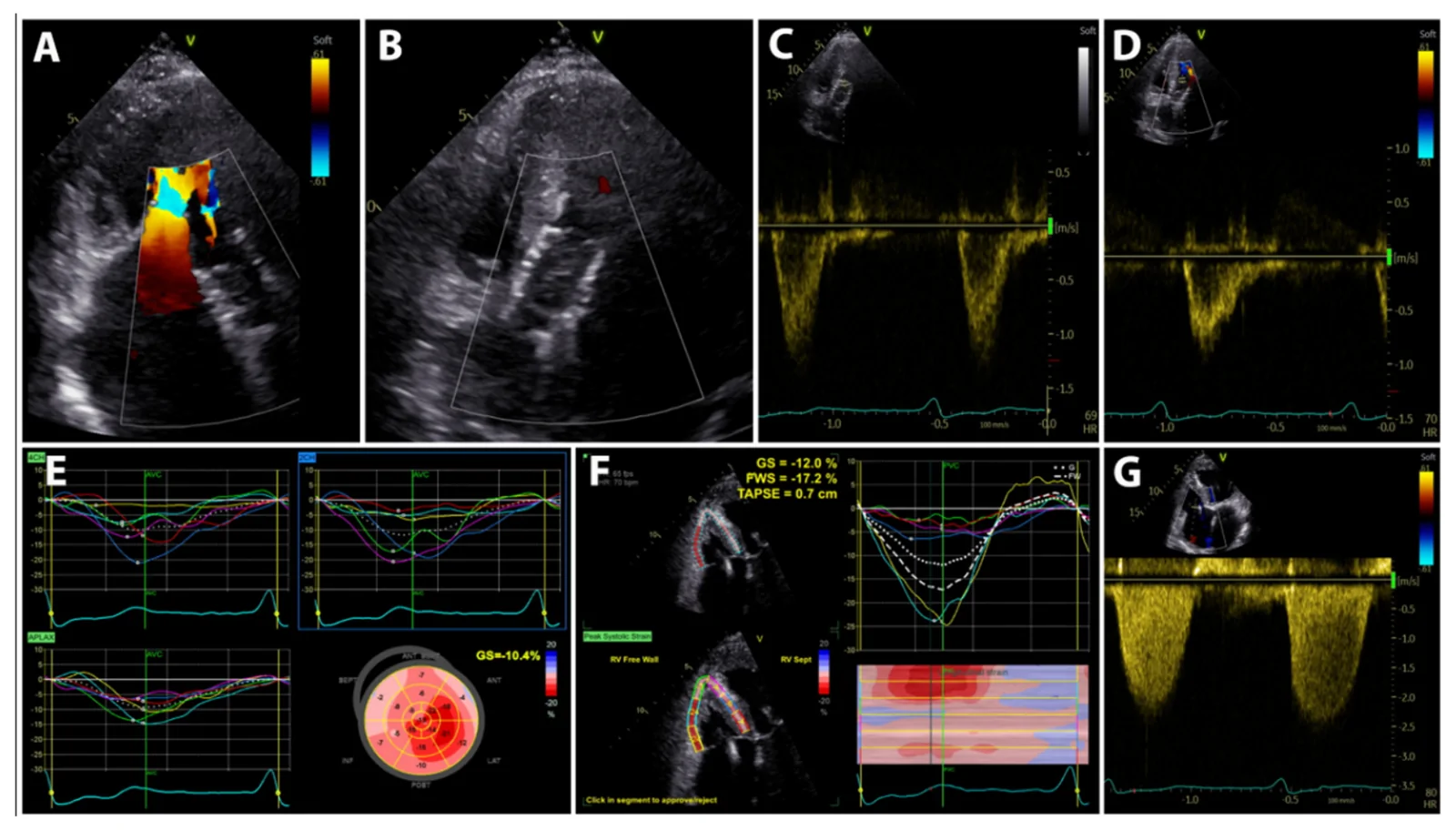
Echocardiographic 1-month follow-up after TAVI. TTE demonstrated a normally functioning Edwards SAPIEN 3 prosthesis with no significant residual aortic regurgitation and normal prosthetic valve hemodynamics (**A**–**D**,**G**). Speckle-tracking echocardiography showed persistent impairment of left ventricular global longitudinal strain and right ventricular longitudinal strain despite marked clinical improvement (**E**,**F**).

The patient demonstrated progressive clinical improvement after TAVI. At 1-month follow-up, the patient demonstrated substantial early clinical and hemodynamic improvement following TAVI. TTE showed a normally functioning Edwards SAPIEN 3 prosthesis, with no significant residual aortic regurgitation and satisfactory prosthetic valve hemodynamics. LV and RV systolic function showed early improvement, although speckle-tracking analysis demonstrated persistent impairment of LV global longitudinal strain and RV longitudinal strain. The absence of significant residual AR and the early recovery of ventricular function were consistent with an effective reduction in the chronic volume overload following valve implantation (Figure 7).

**Figure 8:**
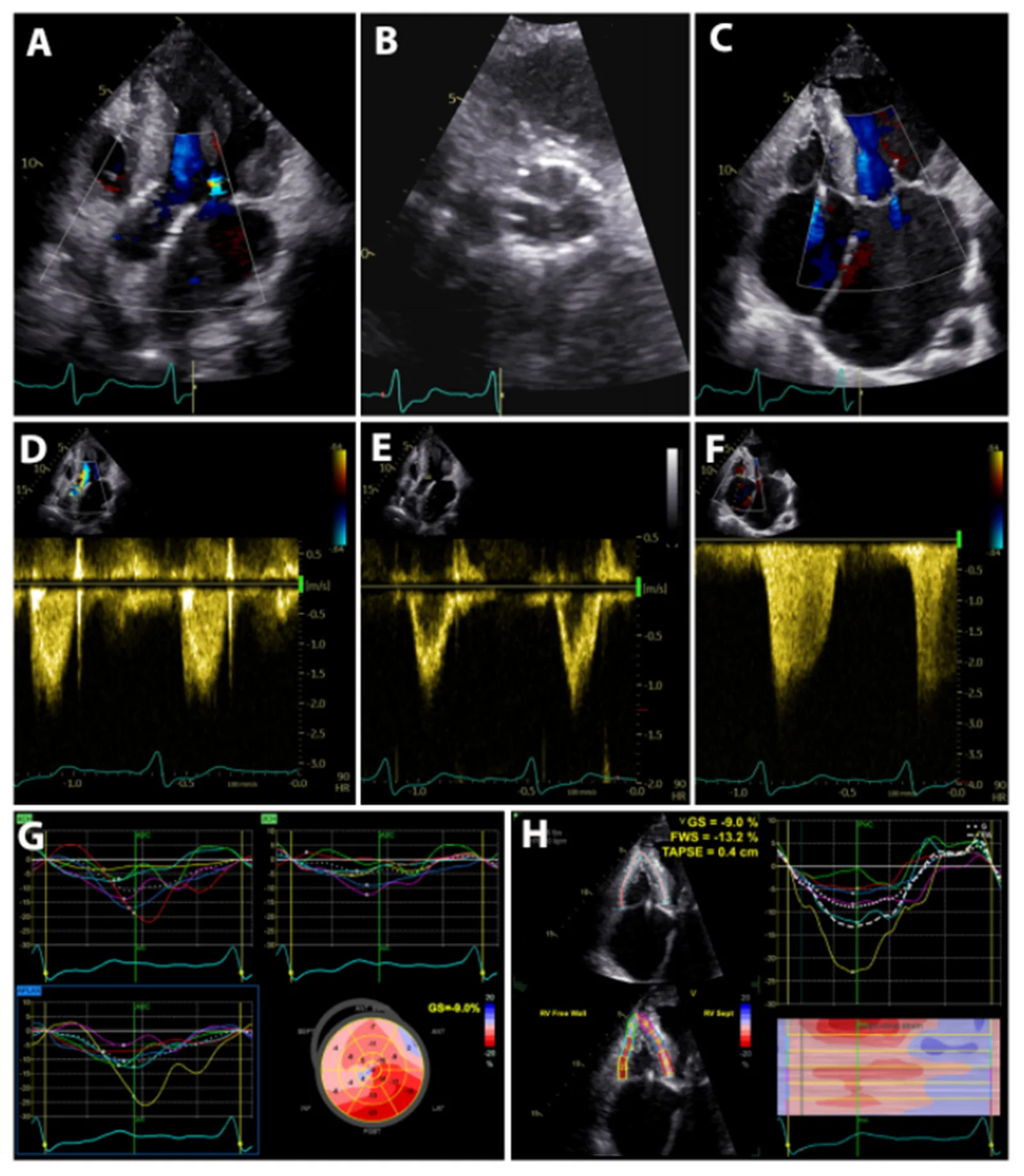
Three-month TTE follow-up after TAVI. (**A**) Apical 5 chambers view color Doppler view shows a normally functioning Edwards SAPIEN 3 prosthesis without significant residual aortic regurgitation. (**B**) Parasternal short-axis view of the prosthetic valve. (**C**) Apical four-chamber color Doppler view demonstrating mild residual mitral and tricuspid regurgitation. (**D**) Pulsed-wave Doppler interrogation of the left ventricular outflow tract demonstrating normal flow velocities. (**E**) Pulsed-wave Doppler at the mid-ventricular septum demonstrating the absence of dynamic left ventricular outflow tract obstruction following septal myectomy and TAVI. (**F**) Continuous-wave Doppler interrogation of the tricuspid regurgitation jet for estimation of pulmonary artery systolic pressure (PASP). (**G**) Left ventricular speckle-tracking analysis demonstrating persistent impairment of global longitudinal strain. (**H**) Right ventricular free-wall strain analysis demonstrates persistent improvement of RV systolic dysfunction.

At 3 months follow-up his functional status had improved substantially, and echocardiographic assessment demonstrated optimal prosthetic valve function and further recovery of ventricular performance. The prosthesis remained well positioned, with no significant residual or paravalvular AR and normal transvalvular flow velocities. There was no evidence of recurrent dynamic LVOT obstruction following septal myectomy and TAVI. Mild residual mitral and tricuspid regurgitation were present, while RV systolic function showed marked improvement, with no echocardiographic evidence of significant pulmonary hypertension. Although LV global longitudinal strain remained impaired, probably in the context of the genetic cardiomyopathy, the overall echocardiographic findings demonstrated progressive biventricular recovery after correction of the severe AR (Figure 8).

Natriuretic peptide levels showed a consistent decline, with NT-proBNP decreasing from 16,933 pg/mL before TAVI to 4044 pg/mL immediately after the procedure. By October 2025 the NT-proBNP level further decreased to 1688 pg/mL, cardiopulmonary exercise testing demonstrated improved exercise tolerance and no significant arrhythmic events were detected by ICD interrogation.

At one-year follow up, the patient reported no dyspnea during ordinary physical activity, no angina or palpitations and good exercise tolerance. He was classified as NYHA functional class I. TTE showed normally functioning prosthesis with mean transvalvular gradient of 7 mmHg, and no paravalvular regurgitation. Left ventricular EF was calculated 59% with no signs of LV outflow obstruction mild-to-moderate mitral regurgitation and mild tricuspid regurgitation were noted, with a normalized RV systolic function and no echocardiographic signs of pulmonary hypertension. Six-minute walk testing revealed 82% of predicted performance with minimal symptoms (Borg dyspnea score 1/10). NT-proBNP had further decreased to 488 pg/mL. The embolized prosthesis remained clinically asymptomatic, and its position was stable as followed by chest radiography. CT imaging has not been performed during the first year of follow-up, but it remains an important consideration for future assessment during long term follow-up.

## 3. Discussions and Review of the Literature

Transcatheter aortic valve implantation (TAVI) for pure native aortic regurgitation (AR) remains an off-label and technically challenging application of transcatheter valve therapy, largely because the absence of annular calcification limits prosthetic anchoring and increases the risk of valve migration or embolization. Although accumulating experience with conventional transcatheter heart valves and the emergence of dedicated devices have improved procedural outcomes, the available evidence remains predominantly observational and is largely derived from elderly patients at high surgical risk.

Against this background, the present case represents a particularly unusual extension of the existing experience with off-label TAVI for native AR. The patient was exceptionally young and had severe AR developed after previous transaortic septal myectomy, a surgically altered and anatomically constrained aortic root, progressive biventricular dysfunction with cardiogenic shock and multiorgan failure, and prohibitive risk for redo surgery. The case was further complicated by initial valve embolization, followed by successful implantation using an individualized bailout strategy. Thus, beyond illustrating the feasibility of off-label TAVI in native AR, this case adds to the limited literature by highlighting the complex interaction between prosthetic anchoring, prior aortic surgery, challenging root anatomy, and lifetime valve management in a young patient.

### 3.1. HCMO–Genotype–Phenotype Correlation

HCM is the most common inherited cardiomyopathy, with an estimated prevalence of 1 in 500 individuals, and is characterized by unexplained left ventricular hypertrophy, myocyte disarray, and interstitial fibrosis [1,2]. In approximately 40–60% of cases, a pathogenic variant in one of the sarcomeric protein genes is identified, with *MYH7* (encoding β-myosin heavy chain) and *MYBPC3* (encoding myosin-binding protein C) together accounting for the majority of genotype-positive cases [3,4]. *MYH7* variants, particularly those affecting the myosin head domain, are generally associated with an earlier age of onset, more pronounced hypertrophy, and a higher risk of heart failure and sudden cardiac death compared with *MYBPC3* variants, which often exhibit later-onset disease with incomplete penetrance [5]. Among *MYH7* mutations, the p.Arg719Trp missense variant in the myosin head domain is one of the most extensively characterized, and it has been linked to a malignant phenotype with high disease penetrance and a particularly elevated risk of lethal arrhythmic events [5,6].

The present case exemplifies several distinctive features of this genotype–phenotype relationship. The patient’s remarkably aggressive family history—with multiple sudden cardiac deaths across three generations, including a sibling who died at 22 despite prophylactic ICD implantation, and an affected 11-year-old niece—underscores the near-complete penetrance and extreme arrhythmic risk conferred by this specific variant. The early disease onset (diagnosis at 13 years, transaortic septal myectomy at 14 years) and the severity of LVOT obstruction requiring surgical intervention in adolescence illustrate the full clinical spectrum of this aggressive sarcomeric disease. Notably, the mitral valve apparatus was also involved—a well-recognized feature of HOCM in which systolic anterior motion of the mitral valve contributes to dynamic LVOT obstruction and is frequently accompanied by intrinsic leaflet elongation and anomalous chordal insertions [7,8]. Concomitant mitral valve repair with secondary chordal resection was performed at the time of myectomy, reflecting the frequent need to address both the septal and mitral components of the obstructive substrate to achieve complete relief of outflow tract obstruction [8]. This case reinforces the importance of comprehensive genetic testing and cascade family screening, as recommended by current guidelines [7].

### 3.2. Aortic Regurgitation After Septal Myectomy: Incidence and Mechanisms. Treatment

Iatrogenic AR following transaortic septal myectomy is an uncommon but recognized complication. In the largest series to date, Juarez-Casso et al. reported procedure-related AR in 2% of 2807 patients undergoing transaortic septal myectomy for HOCM, with 1% requiring unplanned aortic valve procedures at the time of surgery [9]. The cumulative incidence of late aortic valve reoperation was 1% at 10 years and 5% at 15 years. Two distinct mechanisms have been described: acute intraoperative cusp laceration or avulsion, typically identified and repaired during the index procedure, and delayed cusp restriction due to progressive fibrosis and retraction of leaflet tissue, which may become clinically evident years after surgery. The right coronary cusp appears particularly vulnerable owing to its proximity to the septal resection site. The likely pathophysiology involves progressive fibrosis and retraction of the cusp tissue, possibly related to thermal injury, mechanical trauma from retractors, or scarring extending from the myectomy site. Over time, this leads to failure of cusp coaptation and development of severe, eccentric AR. The present case illustrates this latter mechanism—severe AR developed approximately six years after myectomy due to progressive fibrosis and restriction of previously injured right coronary cusp with failure of coaptation, rather than acute cusp injury. Surgical aortic valve replacement is the standard treatment for severe AR; The standard treatment for severe aortic regurgitation is surgical aortic valve replacement. However, in the present case, the decision against redo surgery was driven not by the technical challenges of resternotomy, but by the patient’s profoundly compromised clinical status: recurrent episodes of decompensated heart failure and cardiogenic shock, severe biventricular dysfunction, pulmonary hypertension, congestive hepatopathy with cholestatic jaundice, acute kidney injury, and markedly elevated lactate levels. This multiorgan dysfunction, in the setting of advanced heart failure physiology, rendered the patient an extreme-risk surgical candidate in whom cardiopulmonary bypass and cardioplegic arrest carried a prohibitive risk of perioperative mortality and failure to wean from mechanical circulatory support. Accordingly, after multidisciplinary Heart Team deliberation, transfemoral TAVI with a balloon-expandable valve was chosen as a rescue strategy. Although TAVI remains off-label for pure native aortic regurgitation, its less invasive profile—avoiding sternotomy, cardiopulmonary bypass, and the systemic inflammatory response associated with prolonged cardiac surgery—made it the most appropriate option in this hemodynamically fragile patient [10,11].

### 3.3. TAVI for Pure Native Aortic Regurgitation—Review of the Literature

TAVI for pure native AR remains an off-label indication worldwide. The historical standard of care is surgical aortic valve replacement; however, in patients with prohibitive surgical risk, TAVI has emerged as a viable alternative [10,11].

A review of the literature was conducted to identify studies evaluating TAVI for pure native AR, with particular emphasis on device type, procedural outcomes, complications, and mortality. A comprehensive search of PubMed database was performed through July 2026 using combinations of the following keywords: “aortic regurgitation,” “pure native aortic regurgitation,” “transcatheter aortic valve implantation,” “transcatheter aortic valve replacement,” “TAVI,” “TAVR.” Keywords within the same category were combined with “OR”, whereas keywords across categories were combined with “AND”. Studies reporting clinical outcomes of TAVI for pure native AR, including observational studies, registries, prospective investigations, and relevant case series (>20 cases), were considered for inclusion. Review articles, editorials, conference abstracts, duplicate publications, and overlapping patient populations were excluded to avoid bias selection. Data were independently extracted by two investigators and discrepancies were resolved by consensus with a third reviewer. A total of 371 articles were identified through the initial search. After applying the inclusion and exclusion criteria, 28 articles were selected for review [11,12,13,14,15,16,17,18,19,20,21,22,23,24,25,26,27,28,29,30,31,32,33,34,35,36,37,38]. Data regarding device type and success, procedural complications, mortality, and follow-up outcomes were collected and structured into table for comparative analysis (Table 1).

The studies summarized in Table 1 illustrate the rapid evolution of transcatheter treatment for pure native AR over the past decade. The 28 included studies, published between 2013 and 2026, comprised a total of 3282 patients and consisted predominantly of observational, multicenter, and registry-based investigations, with more recent evidence also including prospective studies and pivotal trials. Notably, the volume of evidence increased substantially over time, with 18 of the 28 studies (64.3%) published from 2023 onward, reflecting growing clinical interest and technological progress in this challenging indication. Conventional transcatheter heart valves used off-label for native AR were evaluated in 17 studies (60.7%), dedicated AR devices in 10 (35.7%), while one study (3.6%) directly compared dedicated and off-label platforms. Reported device or technical success ranged from approximately 72% to 100%, with contemporary studies generally achieving rates above 85–90%, particularly with dedicated devices. The most frequently reported complications were valve embolization/migration, need for a second valve, permanent pacemaker implantation, and residual moderate or greater AR; however, an overall complication rate cannot be reliably calculated because definitions and reporting differed substantially among studies. Early mortality, generally assessed at 30 days, ranged from 0% to 23%, with lower rates observed in several contemporary series. Late mortality ranged from 0% to approximately 20% in most studies reporting 1-year or longer outcomes, although direct comparison is limited by markedly different follow-up durations, extending from 6 months to 5 years. Taken together, these data demonstrate a clear temporal trend toward higher procedural success and improved early outcomes, paralleling advances in device design, implantation strategies, and the introduction of dedicated transcatheter systems for non-calcified native AR.

The largest early series, by Roy et al., examined 43 patients treated with the CoreValve device for pure AR and reported a 30-day mortality of 9.3% and a VARC-defined procedural success of only 74.4%, reflecting the frequent need for a second valve and residual AR [12]. An Italian multicenter registry of 26 patients treated with CoreValve found a 30-day mortality of 23% for pure AR, significantly higher than the 5.9% observed in patients treated for aortic stenosis [13].

More recently, a large contemporary registry of new-generation devices in pure AR (EuroIntervention, 2026) reported VARC-3 technical success of 85.5%, with ≥grade III residual AR in only 1.2% and a 4-year mortality of 53.5% [31]. Device size ≥29 mm was used in 73% of patients, and the need for a second valve occurred in approximately 8–10% of cases irrespective of whether the device was balloon-expandable or self-expanding. The permanent pacemaker implantation rate was 36%, reflecting the aggressive oversizing strategy often employed in non-calcified anatomy.

Balloon-expandable valves (BEVs) have traditionally been considered less suitable for pure native AR due to the absence of annular calcification required for anchoring. However, recent evidence supports their feasibility in selected patients. The French multicenter S3AR study evaluated the SAPIEN 3 transcatheter heart valve in 37 patients with pure AR on non-calcified native valves who were contraindicated for surgery [21]. The device success rate was 94.6%, with 30-day all-cause mortality of 8.1% and valve migration occurring in 10.8% of cases. At 1 year, all-cause mortality was 16.2%, and 89.7% of survivors were in NYHA class ≤II with AR grade ≤2. These results suggest that TAVI with the SAPIEN 3 platform is technically feasible in high-risk patients with pure AR, although the risk of valve migration remains a concern.

A novel technique employing the “flare the outflow” approach for BEVs in pure AR was recently described in a proof-of-concept study [39]. This single-center study of 6 inoperable patients demonstrated 100% procedural success with no valve migration or severe paravalvular leak, using 2 mm oversizing and a deliberate technique to flare the ventricular outflow portion of the frame to enhance anchoring. The 1-year follow-up was satisfactory, though larger studies are needed to confirm durability.

Self-expanding valves (SEVs) have been the most extensively studied platform for pure AR. The STS/ACC TVT Registry analysis included 230 patients who underwent TAVI for primary severe native AR using commercially available self-expanding valves (CoreValve, n = 81; Evolut R, n = 149) between 2014 and 2017 [17]. All patients had moderate/severe AR at baseline. At 30 days, 9.1% of patients continued to have moderate and 1.4% had severe AR. There was a significant reduction in residual moderate/severe AR from the CoreValve to the Evolut R device (19.1% vs. 6.3%, *p* = 0.02), suggesting improvements with newer-generation devices. Despite higher 30-day all-cause mortality compared with the general TAVI population, self-expanding TAVI was considered a viable option in selected patients with AR who had no surgical alternatives.

The PANTHEON International Project is the largest head-to-head comparison of BEVs and SEVs in pure native AR, retrospectively including 201 patients (132 SEV, 69 BEV) from multiple centers [26]. Technical success was comparable between groups, with no significant difference in 30-day mortality (5.3% SEV vs. 4.4% BEV, *p* = 0.767), residual moderate or greater AR (9.2% vs. 10.1%, *p* = 0.835), or valve embolization/migration. The study concluded that both device types are viable options for pure AR, with the choice determined by individual anatomical considerations.

Balloon-expandable valves have traditionally been considered less suitable for pure AR because of the absence of annular calcification for anchoring, unpredictable deployment behavior, and the theoretical risk of annulus rupture with oversizing [10]. Self-expanding devices with broader radial force, retrievability, and dedicated anchoring mechanisms have generally been preferred. However, the SAPIEN 3 platform offers precise positioning, a sealing skirt that may reduce paravalvular leak, and—with adequate oversizing—can achieve stable anchoring even in a non-calcified annulus.

The present case demonstrates that a balloon-expandable device can be used successfully in a non-calcified annulus, though the embolization of the first prosthesis underscores the technical challenge. The decision to use a 29 mm SAPIEN 3 in a relatively small annulus with a narrow root reflects a deliberate oversizing strategy to maximize anchoring surface area. The embolization of the first valve into the descending thoracic aorta—a complication with an incidence of approximately 8–10% in the pure AR TAVI population—highlights the importance of having a second device immediately available and being prepared for this contingency [31].

The limitations of current-generation TAVI devices for pure AR have driven the development of dedicated transcatheter heart valves with anchoring mechanisms that do not depend on annular calcification. Dedicated devices, such as the J-Valve system, which uses claspers to anchor onto the native aortic leaflets, has demonstrated excellent 5-year clinical and echocardiographic outcomes in a multicenter prospective study [31]. The JenaValve Trilogy system similarly employs a dedicated mechanism for non-calcific anatomy and has received both CE mark approval and FDA approval for this indication. These devices may expand the treatment options for patients like the one presented here and may eventually become the standard of care for transcatheter treatment of pure AR. The limitations of off-label conventional TAVI devices in pure AR have driven the development of dedicated transcatheter heart valves. The J-Valve system (JieCheng Medical) uses a unique anchoring mechanism with three U-shaped claspers that engage the native aortic leaflets, providing stable fixation independent of annular calcification. Wang et al. reported 5-year outcomes in a multicenter prospective study, demonstrating excellent clinical and echocardiographic results with this dedicated device [33].

The JenaValve Trilogy (JenaValve Technology) represents the first FDA-approved dedicated transcatheter heart valve for high-risk patients with symptomatic severe AR [40]. In over 700 patients with aortic regurgitation, the Trilogy system achieved 95% technical success with only 0.5% ≥ moderate AR at 30 days. The ALIGN-AR pivotal trial demonstrated durable outcomes at 2-year follow-up, supporting its role as a dedicated on-label option for this challenging population [41]. These dedicated devices represent a paradigm shift and may expand transcatheter treatment to a broader AR population in the future.

### 3.4. Right Ventricular Dysfunction and Multiorgan Recovery

Severe AR imposes a unique hemodynamic burden characterized by chronic left ventricular volume overload, which, if left uncorrected, initiates a cascade of secondary downstream effects [42]. Elevated left ventricular end-diastolic pressure is transmitted retrogradely to the left atrium, the pulmonary venous circulation, and ultimately the right ventricle, producing post-capillary pulmonary hypertension and progressive right ventricular dysfunction [43]. Systemic venous congestion ensues, with the potential to compromise hepatic, renal, and splanchnic function—a sequence collectively recognized as cardiohepatic and cardiorenal syndromes in the setting of valvular heart disease [43].

One of the most challenging clinical questions in patients with severe valvular heart disease and advanced multiorgan dysfunction is whether the observed end-organ damage represents irreversible parenchymal injury or is a reversible consequence of the valvular lesion itself [44]. In the former scenario, the risks of intervention may outweigh its benefits; in the latter, definitive correction of the valvular abnormality can produce dramatic recovery. Distinguishing between these two possibilities is often possible only by observing the response to intervention—a decision that requires clinical judgment and, as in this case, multidisciplinary consensus.

The development of severe aortic regurgitation in this patient triggered a predictable but profound hemodynamic cascade. Chronic LV volume overload led to progressive left ventricular dilatation, elevated left atrial pressure, and post-capillary pulmonary hypertension, which in turn increased right ventricular afterload and ultimately resulted in severe right ventricular dysfunction. The ensuing systemic venous congestion produced the full spectrum of multiorgan involvement observed in this case: congestive hepatopathy with marked cholestatic jaundice, acute kidney injury, and recurrent episodes of cardiogenic shock with lactic acidosis. The critical question—whether these abnormalities reflected irreversible end-organ damage or were primarily secondary to the valvular lesion—could only be answered by correcting the underlying hemodynamic insult. The response to TAVI provided a definitive answer: within months, right ventricular function normalized, pulmonary hypertension resolved completely, hepatic and renal parameters returned to baseline, and NT-proBNP normalized. This reduction underscores the concept that the multiorgan dysfunction in this setting was predominantly afterload-dependent and reversible, representing cardiohepatic and cardiorenal syndromes driven by valvular heart disease rather than intrinsic parenchymal failure. The case reinforces the principle that even profound multiorgan dysfunction should not preclude definitive valvular intervention when the valvular lesion is the identifiable and correctable driver of decompensation.

### 3.5. TAVI in Young Patients: Durability and Lifetime Management

Bioprosthetic valves, whether implanted surgically or via a transcatheter approach, are inherently subject to structural valve deterioration (SVD)—a process driven by progressive leaflet calcification, tissue degeneration, and mechanical wear [45]. Current-generation surgical bioprostheses have a median durability of approximately 10–15 years [46], while clinically relevant SVD in transcatheter valves emerges as early as 5–8 years, with younger patient age being one of the strongest independent predictors of accelerated degeneration [47,48].

The implantation of a transcatheter bioprosthesis in a 24-year-old patient raises critical questions regarding long-term durability and lifetime management. The use of RESILIA tissue—a bovine pericardial tissue treated with an advanced anti-calcification process—was a deliberate choice in this context, as 10-year COMMENCE trial data have demonstrated 97.3% freedom from SVD with RESILIA tissue versus 90.5% with conventional bioprostheses [49,50]. Although RESILIA tissue has demonstrated encouraging mid- to long-term durability in surgical bioprostheses, these data cannot be directly extrapolated to transcatheter implantation in very young patients, in whom the lifetime exposure to hemodynamic and biological stress is substantially greater and very long-term TAVI durability data remain limited. Even with enhanced tissue durability, SVD is an expected long-term event given the patient’s age. The 29 mm prosthesis provides a favorable internal diameter and may facilitate future valve-in-valve TAVI and coronary re-access during lifetime management.

The need for repeated valve interventions over the patient’s lifetime remains an important consideration. Given his young age, structural valve deterioration of the current bioprosthesis would be expected to occur within his lifetime, potentially necessitating subsequent valve-in-valve TAVI and, eventually, surgical valve replacement or transcatheter valve explantation. Each additional intervention may progressively increase procedural complexity, risk of patient–prosthesis mismatch, coronary access limitations, and the challenges of future surgical explantation. Therefore, the current TAVI should be regarded as one step within a lifetime management strategy rather than a definitive treatment. In the longer term, if repeated valve interventions become no longer feasible or if progressive ventricular dysfunction or HOCM-related heart failure develops despite optimal treatment, advanced heart failure therapies, including heart transplantation, may ultimately be considered.

Importantly, the marked recovery of biventricular function and end-organ function achieved after TAVI has transformed the patient’s clinical trajectory: should definitive surgical aortic valve replacement, surgical explantation of the transcatheter valve, or even heart transplantation become necessary in the future, these interventions could potentially be undertaken under more favorable clinical conditions. Crucially, however, the underlying HOCM persists irrespective of valvular correction, and lifelong multidisciplinary surveillance for disease progression, arrhythmic risk, and prosthetic valve function remains mandatory.

## 4. Limitations

This report is limited by its single-patient design, and its findings cannot be generalized to the broader population of patients with pure AR or hypertrophic obstructive cardiomyopathy. The favorable outcome reflects careful multidisciplinary patient selection and may not be reproducible in all high-risk cases. The off-label use of a balloon-expandable transcatheter valve in pure AR is supported by limited evidence, but long-term durability, hemodynamic performance, and the optimal lifetime management strategy in young patients remain uncertain. The absence of a dedicated transcatheter device for pure AR at the time of the procedure influenced the therapeutic choice and may not reflect evolving practice. Finally, although the patient’s marked clinical improvement suggests that correction of severe AR was the principal driver of acute recovery, the natural history of HOCM and the risk of heart failure progression mandate long-term surveillance, and whether TAVI ultimately serves as a bridge to heart transplantation or as long-term destination therapy in this young patient remains to be determined.

## 5. Conclusions

Severe aortic regurgitation caused by progressive cusp restriction is a rare late complication of septal myectomy that warrants long-term surveillance in patients with hypertrophic obstructive cardiomyopathy. This case demonstrates how post-myectomy aortic regurgitation can drive a progressive cascade of left ventricular volume overload, worsening heart failure with recurrent episodes of decompensation, and ultimately cardiogenic shock complicated by severe biventricular dysfunction and multiorgan failure. In carefully selected patients with prohibitive surgical risk, transfemoral TAVI may represent an effective rescue strategy capable of restoring hemodynamic stability and promoting sustained ventricular and end-organ recovery. Furthermore, the procedural experience, including intentional oversizing, management of valve embolization, and a modified implantation technique, provides practical insights into the technical challenges of TAVI in non-calcified native aortic regurgitation. Although dedicated transcatheter devices are likely to redefine the treatment of pure native aortic regurgitation, this case supports the use of conventional transcatheter valves as a feasible alternative when no other therapeutic option exists, emphasizing the importance of individualized Heart Team decision-making.

## Figures and Tables

**Table 1:** Key Studies of TAVI for Pure Native Aortic Regurgitation by Device Type.

	Study	Study Type	Year	No.	Device Type	Device Specifics	Device Success	Complications	Early Mortality	Late Mortality
**1**	Roy et al. [12]	Multicenter	2013	43	Off-label device	CoreValve SEV	74.4%	2nd valve 18.6%	9.3% (30 d)	NA
**2**	Testa et al. [13]	Multicenter	2014	26	Off-label device	CoreValve SEV	NA	2nd valve 23.1%; 11.5% PPI	23.0% (30 d)	NA
**3**	Seiffert et al. [14]	Observational	2014	31	Dedicated device	Jena valve	97%	≥mod AR 9%	12.9% (30 d)	19.3% (6 m)
**4**	Yoon et al. [11]	Int’l Registry	2017	331	Off-label device	SEV + BEV (NGD)	81.1%	≥mod AR 4.2%	10.9% (30 d)	NA
**5**	Sawaya et al. [15]	International	2017	78	Off-label device	SEV + BEV (NGD)	72%	2nd valve 47%	14% (30 d)	NA
**6**	Liu et al. [20]	Observational	2018	43	Dedicated device	J-valve	97.7%	0%	2.3% (30 d)	4.7% (1-y)
**7**	Anwaruddin et al. [17]	US Registry	2019	230	Off-label device	CoreValve/Evolut R SEV	NA	≥mod AR 10.5% PPI 22%	13.3% (30 d)	NA
**8**	Shi et al. [18]	Multicenter	2020	44	Dedicated device	J-valve	91.5%	≥mod AR 1.5%	0%	0%
**9**	Yin et al. [19]	Observational	2022	25	Off-label device	SEV + BEV (NGD)	100%	≥mod AR 11.8% PPI 35.3%	13%	20%
**10**	Liu et al. [20]	Observational	2022	134	Dedicated device	J-valve	97.1%	2nd valve 3.7%;	0%	7.4% (1-y)
**11**	Delhomme et al. [21]	Multicenter	2023	37	Off-label device	BEV (NGD)	94.6%	10.8% TVEM	8.1% (30 d)	16.2% (1-y)
**12**	Polleti et al. [26]	International	2023	201	Off-label device	SEV (66%) + BEV (34%) (NGD)	83.6% BEV 76.1% SEV	≥mod AR 9.5% TVEM 8.5%	5.0% (30 d)	17.1% (1-y)
**13**	Zheng et al. [23]	Observational	2023	45	Off-label device	VenusA valve SEV	97.8%	2nd valve 2.2%	0% (30 d)	4.7% (1-y)
**14**	Garcia et al. [24]	Observational	2023	27	Dedicated device	J-Valve	81%	4% TVEM 13% PPI	4% (30 d)	12% (1y)
**15**	Vahl et al. [25]	Pivotal Trial	2024	180	Dedicated device	JenaValve Trilogy	95%	≥mod AR 0.5%(30 d) 24% PPI	2% (30 d)	7.8% (1y)
**16**	Poletti et al. [22]	Multicenter	2024	256	Dedicated vs. Off-Label	Dedicated vs. Off-Label	87%	Dedicated devices superior	6.6% Off-label vs. 1.1% dedicated(30 d)	14.4% Off-label 17.2% dedicated (1-y)
**17**	Mao et al. [27]	Observational	2024	598	Dedicated device	J-valve	95.8%	8.7% PPI 0.2% 2nd valve	NA	NA
**18**	Lin et al. [28]	Observational	2024	103	Off-label device	VenusA/VitaFlow valve SEV	100%	PPI 20% 2nd valve 0%	0%	0%
**19**	Hinkov et al. [29]	Observational	2024	27	Off-label device	SEV + BEV (NGD)	100%	5.2% PPI 2nd valve 22%	3.7% (30 d)	25%
**20**	Kong et al. [30]	Observational	2024	62	Off-label device	VitaFlow valve	79%	2nd valve 1.6% 29% PPI	1.6% (30 d)	6.5% (1-y)
**21**	Le Ruz et al. [31]	Registry	2024	227	Off-label device	SEV + BEV (NGD)	85.5%	2nd valve ~8–10%; PPI 36%; ≥grade III AR 1.2%	8.4% (30 d)	53.5% (4-yr)
**22**	Kumar et al. [32]	Multicenter	2025	104	Off-label device	SEV + BEV (NGD)	79.8%	8.6% TVEM	1.2% (30 d)	11.8% (1-y)
**23**	Wang et al. [33]	Multicenter	2025	36	Dedicated device	J-valve	NA	NA	NA	15.3% (5-y)
**24**	Yang et al. [34]	Observational	2025	87	Off-label device	VenusA/VitaFlow valve	92.9% vs. 73.3%	PPI 13.0 vs. 34.1% 2nd valve 13.0% vs. 29.3%	4.3%vs 7.3% (30 d)	NA
**25**	Yu et al. [35]	Observational	2025	100	Off-label device	VitaFlow valve	91%	PPI 9% 2nd valve 9%	0%	NA
**26**	Zhu et al. [36]	Observational	2025	47	Dedicated device	J-valve	100%	2nd valve 0%	0%	0%
**27**	Pan et al. [37]	Multicenter	2025	128	Dedicated device	Hancor valve	96.1%	PPI 12% 2nd valve 1.6%	2.3% (30 d)	NA
**28**	Ielasi et al. [38]	Observational	2026	32	Off-label device	Myval BE THV	87.5%	12.5% TVEM 9.4% PPI	0% (30 d)	3.1% (1-y)

Abbreviations: AR—aortic regurgitation, BEV—balloon expandable valve, NGD—new generation devices, PPI—pacemaker implantation, SEV—self-expanding valve, TVEM—transcatheter embolization or migration. NA—not available.

## Data Availability

No new data were created or analyzed in this study.
